# Aqueous Extract of Guava (*Psidium guajava* L.) Leaf Ameliorates Hyperglycemia by Promoting Hepatic Glycogen Synthesis and Modulating Gut Microbiota

**DOI:** 10.3389/fphar.2022.907702

**Published:** 2022-06-01

**Authors:** Shuzhou Chu, Feng Zhang, Huiying Wang, Lijun Xie, Zhinan Chen, Weimin Zeng, Zhiguang Zhou, Fang Hu

**Affiliations:** ^1^ National Clinical Research Center for Metabolic Diseases, Key Laboratory of Diabetes Immunology (Central South University), Ministry of Education, Metabolic Syndrome Research Center, Department of Metabolism and Endocrinology, The Second Xiangya Hospital of Central South University, Changsha, China; ^2^ Key Laboratory of Biometallurgy, School of Minerals Processing and Bioengineering, Ministry of Education, Central South University, Changsha, China

**Keywords:** guava leaf, hyperglycemia, liver, glucose metabolism, gut microbiota

## Abstract

Type 2 diabetes mellitus (T2DM) is a major global health concern. *Psidium guajava* L. (guava) is widely used for food as well as a folk medicine. Previous studies have shown its anti-diabetic and anti-inflammatory properties. However, the underlying mechanisms remains to be elusive. In this study, we assessed the potential therapeutic effects of aqueous extract of guava leaves (GvAEx) on T2DM and explored their potential mechanisms *in vivo* and *in vitro*. GvAEx was gavage administered for 12 weeks in diabetic db/db mice. Our results have demonstrated that GvAEx significantly lowered fasting plasma glucose levels (*p* < 0.01) and improved glucose tolerance and insulin sensitivity (*p* < 0.01, *p* < 0.05, respectively). Additionally, GvAEx increased hepatic glycogen accumulation, glucose uptake and decreased the mRNA expression levels of gluconeogenic genes. Furthermore, GvAEx-treatment caused higher glucose transporter 2 (GLUT2) expression in the membrane in hepatocytes. Notably, for the first time, we have elaborated the possible mechanism of the hypoglycemic effect of GvAEx from the perspective of intestinal microbiota. GvAEx has significantly changed the composition of microbiota and increased short chain fatty acid (SCFA) -producing Lachnospiraceae family and *Akkermansia* genus in the gut. Taken together, GvAEx could alleviate hyperglycemia and insulin resistance of T2DM by regulating glucose metabolism in the liver and restoring the gut microbiota. Thus, GvAEx has the potential for drug development against T2DM.

## Introduction

Diabetes mellitus (DM) is one of the most prevalent metabolic diseases in the world. As of 2019, an estimated 463 million people worldwide have DM (8.8% of the adult population), with T2DM accounting for approximately 90% of cases (Cho et al., 2018). That number is expected to reach 643 million by 2030 and 783 million by 2045. Direct medical expenditures related to diabetes are already approaching one trillion dollars and will exceed that figure by 2030 (IDF, 2019). DM is caused by either the pancreas not producing enough insulin, or the body’s cells not responding appropriately to the effects of insulin (DeFronzo et al., 2015).

Insulin resistance is recognized as an essential risk factor for the development of T2DM. There is an impaired biological response to insulin stimulation in target tissues, mainly liver, muscle and adipose tissue (Wild et al., 2004). The liver plays a key role in glucose homeostasis by regulating different pathways of glucose metabolism, including glycolysis, gluconeogenesis, glycogenolysis sand glycogenesis (Han et al., 2016). Due to insulin resistant, hepatic glycogen synthesis is reduced while gluconeogenesis is increased, which eventually leads to the increased rate of hepatic glucose output and causes hyperglycemia (Petersen et al., 2017). Accordingly, it is important to identify new approaches to improve hepatic insulin sensitivity against T2DM. The therapeutic properties of some plants have long been recognized for hundreds and thousands of years. However, these herb medicines are used as mixtures or concentrated extracts without isolation of active compounds. Recent years, the development of advanced technologies have made it possible to extract the active compounds from these plants, which can be used in the pharmaceutical, food and chemical industries (Guo et al., 2021; Tan et al., 2022).

*Psidium guajava* L., also known as guava, is a member of the myrtle family (Myrtaceae), which is cultivated in many tropical and subtropical regions as fruit (Morton, 1987). Not only is guava widely used for food, but is also a folk medicine. Guava leaves are the most important part for medicinal purposes. Traditionally, the guava leaves are used as herbal medicine for the prevention and treatment of diseases. Nowadays, people also use extracts from its fruit, bark or roots for their anti-microbial, hepatoprotective, anti-diabetic and anti-inflammatory properties (Dakappa et al., 2013; Morais-Braga et al., 2016; Díaz-de-Cerio et al., 2017). The profile of secondary plant metabolites of guava includes several phytoconstituents such as various terpenoids, flavonoids, carotenoid and phenolic (Rojas-Garbanzo et al., 2017). Significantly, guava leaf has been reported to protect against T2DM. Guava-leaf aqueous extract (GvAEx) treatment for 6 weeks attenuated the progression of hyperglycemia and hyperlipidemia in diabetic mice (Jayachandran et al., 2020). Besides, GvAEx significantly reduced postprandial glucose level in human subject while had no hypoglycemia side effect (Deguchi and Miyazaki, 2010). It is reported that polysaccharides and flavonoid compounds purified from guava leaves synergistically inhibited α-glucosidase and α-amylase*,* which would delay the absorption of glucose in the small intestine to lower blood glucose levels (Zhang et al., 2016; Beidokhti et al., 2020). Other studies have found that guava leaf extract may exert its anti-diabetic effects by activating the PI3K/AKT signaling pathway in the liver and muscle of diabetic mice (Vinayagam et al., 2018; Jayachandran et al., 2020). Recently, many studies have implied that hypoglycemic drugs can work through the regulation of intestinal flora, and that changes in intestinal flora can, in turn, affect the efficacy of hypoglycemic drugs (Wu et al., 2017; Balaich et al., 2021). However, it remains to be determined on whether GvAEx exerts its anti-diabetic action by regulation of the intestinal flora.

In the present study, we investigated the hypoglycemic effects of GvAEx and explored its underlying mechanism *in vivo* and *in vitro*. This study provides new evidence about the anti-diabetic action of GvAEx. More importantly, for the first time, we elaborated the potential mechanism of the hypoglycemic effect of GvAEx from the perspective of intestinal microbiota.

## Research Design and Methods

### Reagents

Guava leaf aqueous extracts were obtained from our previously reported methods (Bai et al., 2019). The primary antibodies for mouse β-actin, ATP1A1, phospho-AKT (Ser473), AKT, phospho-GSK3β (Ser9), GSK3β were obtained from Cell Signaling Technology Inc. (Beverly, MA, United States). Anti-GLUT2 antibody was purchased from Proteintech (Chicago, United States).

### Animal Studies

Six-week-old male db/db mice were purchased from the National Resource Center for Mutant Mice (Nanjing, Jiangsu, China) and housed in a temperature-controlled environment with a 12:12 h light/dark cycle. The mice had free access to food and water *ad libitum*. After a 1-week adaptive period, the mice were randomly divided into two weight-matched groups (*n* = 8/group) and fed with chow diet (10% kcal; #12540B; Research Diets, Inc., New Brunswick, United States). The body weight and fasting plasma glucose level were monitored weekly. After 12 weeks of gavage administration with GvAEx (7.0 g/kg) or water, animals were sacrificed and their tissues were rapidly isolated, then immediately frozen in liquid nitrogen and stored at −80°C prior for further experiments. All animal experiments were conducted in accordance with the guidelines issued by the Institutional Animal Care and Use Committee (IACUC) of the Second Xiangya Hospital of Central South University, Changsha, Hunan, P. R. China (Approval #202022).

Glucose tolerance test (GTT) and insulin tolerance test (ITT) were performed according to previous reported methods (Kong et al., 2015). For GTT, mice were fasted overnight for about 16 h and glucose was injected intraperitoneally (i.p.) at a dose of 2 g/kg. For ITT, food was removed from mice 4 h prior to experiment and insulin was administered i.p. at 0.4 IU/kg. The caudal vein blood glucose levels were measured at 0, 30, 60, 90, and 120 min after the administration of glucose or insulin. Homeostatic model assessment for insulin resistance (HOMA-IR) index was calculated using the following equation [fasting blood glucose (mmol/L) × fasting insulin (μU/mL)]/22.5.

### Metabolic and Biochemical Analyses

The content of glycogen in liver and muscle was measured according to manufacturer’s protocols (Solarbio Science and Technology Co., Ltd. Beijing, China). Briefly, precipitates of liver and muscle were added 0.2% anthrone diluted with 98% concentrated sulfuric acid (H_2_SO_4_) and followed by boiling water-bath heating for 20 min. After cooling on the ice, the OD values were measured at 620 nm by an ELISA reader. The serum insulin and c-peptide levels were determined using mouse insulin enzyme-linked immunosorbent assay (ELISA) kits (CUSABIO, Wuhan, China) according to manufacturer’s instructions.

Serum lipids (total cholesterol, triglyceride) and functional parameters of liver [alanine aminotransferase (ALT), aspartate transaminase (AST)] and renal (creatinine, urea) were measured using a Modular Analytics analyzer (Roche Diagnostics GmbH, Mannheim, Germany) according to manufacturer’s instructions.

### Cell Culture and Treatment

HepIR mouse liver cells were cultured in MEMα (Gibco) culture medium supplemented with 4% fetal bovine serum (FBS; Gibco) 100 U/ml penicillin and 100 U/ml streptomycin (Gibco) at 37°C. Cells were pre-incubated in serum-free medium for 4 h and then treated with 200 uM PA to induce insulin resistance.

The glucose uptake of HepIR cells was evaluated using the fluorescent glucose 2-[N-(7-nitrobenz-2-oxa-1,3diazol-4-yl) amino]-2-deoxyglucose (2-NBDG) (Cayman Chemical, Ann Arbor, MI, United States). The effect of GvAEx on glucose uptake was assessed in PA induced insulin-resistant HepIR cells. Briefly, PA induced insulin-resistant HepIR cells were treated with GvAEx (0.1–2 mg/ml) or vehicle in glucose-free DMEM for 1 h. After aspirating the supernatant, the cells were incubated with insulin (10 nM) (Solarbio, Beijing, China) or saline for 10 min. The cells were then gently rinsed with HBSS and incubated with 100 ug/mL 2-NBDG at 37°C for 50 min. The cells were washed with HBSS and collected for further analysis. The fluorescence intensity of the cells was detected by a microplate reader (excitation/emission = 465/540 nm). Relative 2-NBDG (%) uptake was calculated using the following equation (intensity of treatment/intensity of normal control) × 100%.

### Membrane and Cytosolic Protein Extraction

Proteins from the membrane and cytoplasm fractions of HepIR cells were isolated using the Membrane and Cytosol Protein Extraction Kit (P0033, Beyotime Biotechnology, China) according to the manufacturer’s instructions. Briefly, about 5 × 10^6^ cells in the culture medium were scrapped off the surface of the plate with a cell scraper. Cell suspension was centrifuged at 600 × g at 4°C for 5 min to precipitate cells. Membrane Protein Extraction Reagent A with Phenylmethanesulfonyl fluoride (PMSF) (ST506, Beyotime) was added in cells. The cells were gently and fully suspended and set in an ice bath for 10–15 min. The homogenate was placed on ice for 15 min, centrifuged at 700 × g for 10 min, then further centrifuged at 14,000 × g for 30 min. The supernatant (cell cytosol) was carefully collected, and the pellet was then re-suspended in Membrane Protein Extraction Reagent B and centrifuged at 14,000 × g for 5 min. Finally, the supernatant containing the cell membrane proteins was collected. Western blotting was performed for membrane and cell membrane proteins, and Na+/K + ATPase α1 and β-actin were used as internal controls for membrane and cytosolic proteins, respectively.

### Cell Viability Analysis

The cytotoxicity of GvAEx on HepIR cells was assessed with Cell Counting Kit-8 (CCK-8, CK04, DOJINDO) according to the manufacturer’s protocol. After cells were co-cultured with GvAEx (0.1–3 mg/ml) at 37°C for 24-h, CCK-8 reagent was added to the culture medium at a ratio of 1:10 for another 1 h. The absorbance value of each well was measured at 450 nm using a microplate reader to determine cell viability. Cell viability (%) = (absorbance of treatment - absorbance of blank well)/(absorbance of control-absorbance of blank well) × 100%.

### Western Blotting

For protein extraction, HepIR cells were homogenized in 100 µL RIPA buffer (P0013B, Beyotime). Proteins were transferred to polyvinylidene difluoride membrane and incubated with a blocking buffer (5% BSA in 20 mM Tris-HCl, pH 7.5, 137 mM NaCl, and 0.1% Tween 20) for 1 h at room temperature. Membranes were incubated with primary antibodies for 16 h at 4°C, washed three times for 10 min each and then incubated with second antibodies for 1 h at room temperature. Signals were detected using the ChemiDoc™ XRS+ and the Image Lab™ system (BIO-RAD, United States).

### Quantitative RT-PCR

Total RNAs were isolated from tissues or cells with the Trizol reagent (Invitrogen, Shanghai, China) following the manufacturer’s protocol. Reverse transcription of mRNA was performed using a Revert Aid First Strand cDNA Synthesis Kit (Thermo Scientific, United States). Real-time polymerase chain reaction was performed using the FastStart universal SYBR Green Master (Roche, United States) on a 7900HT Fast Real-Time PCR System (Applied Biosystems). The standard amplification program in polymerase chain reaction system was used, which consisted of 1cycle at 95°C for 10 mi, followed by 40 cycles of 95°Cdenatured for 30 s, 60°C annealed for 1 min, and72°C elongated for 1 min. Primers were listed in Supplementary Table S1.

### Fecal DNA Extraction and 16s rRNA Gene Sequencing

Total genome DNA from samples was extracted using an Equal bit 1x dsDNA HS Assay Kit (Lot: 7E421E0) based on kit protocols. The specific experimental details used to amplify the 16S rRNA gene targeting the V3-V4 region by PCR were performed as previously described (Jia et al., 2021).

The DNA library concentration was determined using the Qubit 3.0 Fluorometer. DNA libraries were multiplexed and loaded on the Illumina MiSeq/NovaSeq (Illumina, San Diego, CA, United States) platform according to manufacturer’s instructions. Sequencing was performed in pairs and image analysis and base calling were conducted by the control software embedded in the instrument. The 16S rRNA gene sequencing and preliminary data analysis were provided by GENEWIZ Inc. (Suzhou, China). A total of 705,692 reads were obtained, and 612,351 high-quality sequences were obtained after optimizing the sequencing raw data. Among of them, 327,943 reads were obtained from GvAEx group, accounting for 53.6%; 284,408 reads were obtained from control group, accounting for 46.4%. The number of each sample reads ranged from 39,021 to 62,994, with an average length of 459 bp. The analysis of abundance, *a* and β diversity of OTUs were performed by Perl and R language software to obtain information on the species richness and evenness of the samples. Principal co-ordinates analyses (PCoA) were performed to determine the significant microbial community differences between the samples.

### Data Analysis

All results were presented as means ± SEM. Student’s *t* test analysis between two groups was performed with SPSS Statistics Software (version 19.0; IBM Corp., Armonk, New York). *p* < 0.05 was considered statistically significant.

## Results

### GvAEx Treatment Lowers Blood Glucose in a Dosage-Dependent Manner in db/db Mice

**FIGURE 1:**
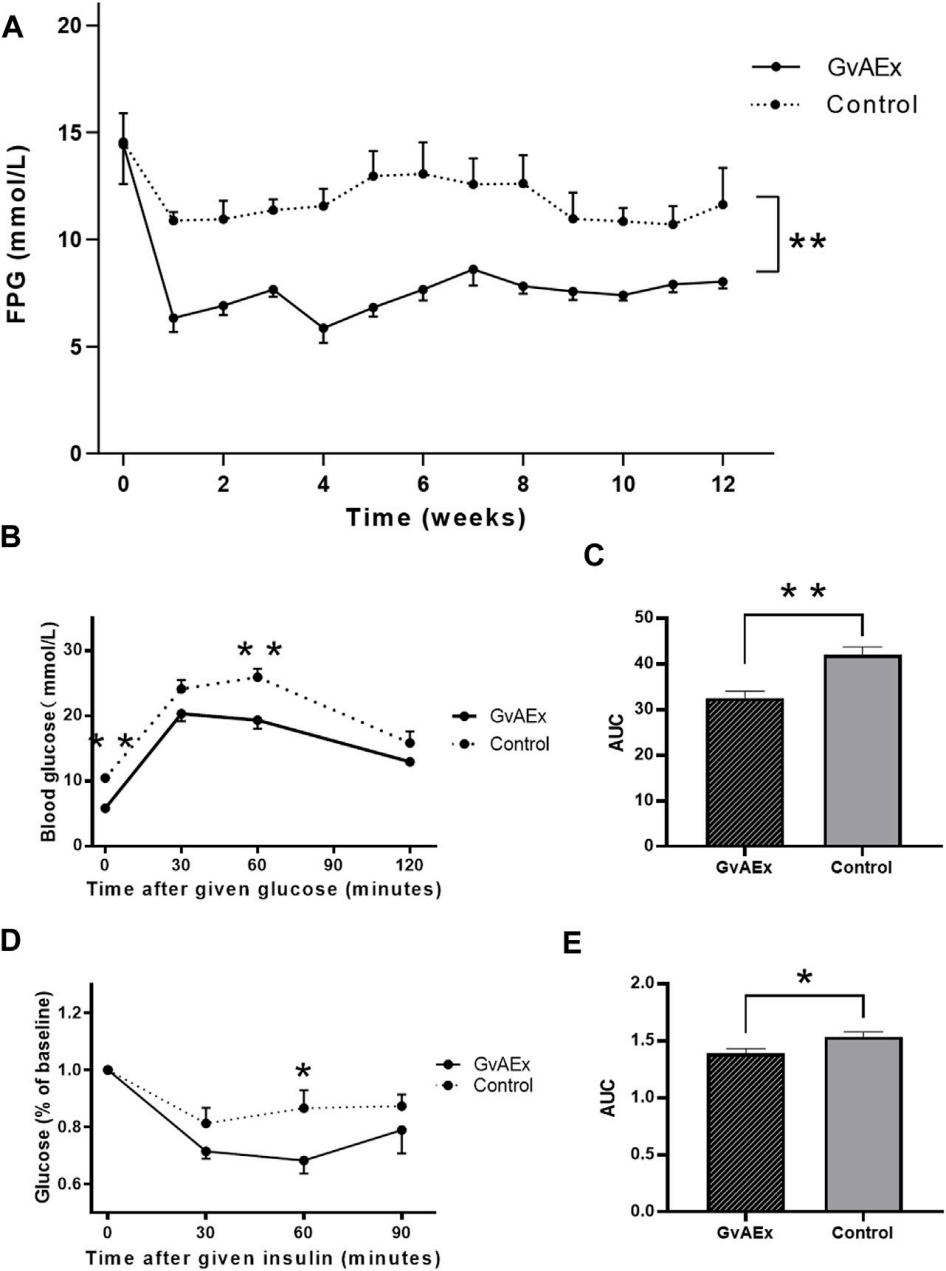
GvAEx treatment lowers blood glucose in a dosage-dependent manner in db/db mice. **(A)** Fasting plasma glucose (FPG) levels of male db/db mice treated with GvAEx or vehicle for 12 weeks (*n* = 6/group). **(B)** Blood glucose levels in the db/db mice treated with GvAEx or vehicle as determined by IPGTT (*n* = 6/group). **(C)** Area under curve (AUC) from **(B)**. **(D)** Blood glucose levels as determined by ITT (*n* = 6/group). **(E)** AUC from **(D)**. *p < 0.05, **p < 0.01, ***p < 0.001.

To explore the effects of GvAEx on the blood glucose, we used three dosages (1.75 g/kg/d, 3.5 g/kg/d, 7.0 g/kg/d) in a preliminary experiment and found that the lowest dose (1.75 g/kg/d) had no effects on fasting plasma glucose (FPG) during 12 weeks of treatment (Supplementary Figure S1A). However, the median dose (3.5 g/kg/d) could reduce blood glucose in diabetic db/db mice after 10 weeks of treatment (Supplementary Figure S1A), and the highest dose (7.0 g/kg/d) had obvious blood-lowering effects starting from the first week of treatment, which could be maintained throughout the 12 weeks treatment period (Figure 1A, *p* < 0.01), indicating a dosage-dependent blood glucose lowering effect of GvAEx in db/db mice. Therefore, we have selected 7.0 g/kg/d as the dosage of GvAEx treatment in the following studies. There were no differences in body weight between GvAEx and control groups (Supplementary Figure S1B).

To determine whether GvAEx had effects on glucose tolerance and insulin sensitivity, we performed GTT and ITT analyses after 12 weeks treatment and found that GvAEx treatment improved glucose tolerance and insulin sensitivity (Figures 1B–E, *p* < 0.01, *p* < 0.05, respectively) in db/db mice. Taken together, the GvAEx treatment had attenuated hyperglycemia and improved glucose tolerance and insulin sensitivity in db/db mice.

### Treatment of GvAEx Improves Kidney and Islet Function in Diabetic Mice

**FIGURE 2:**
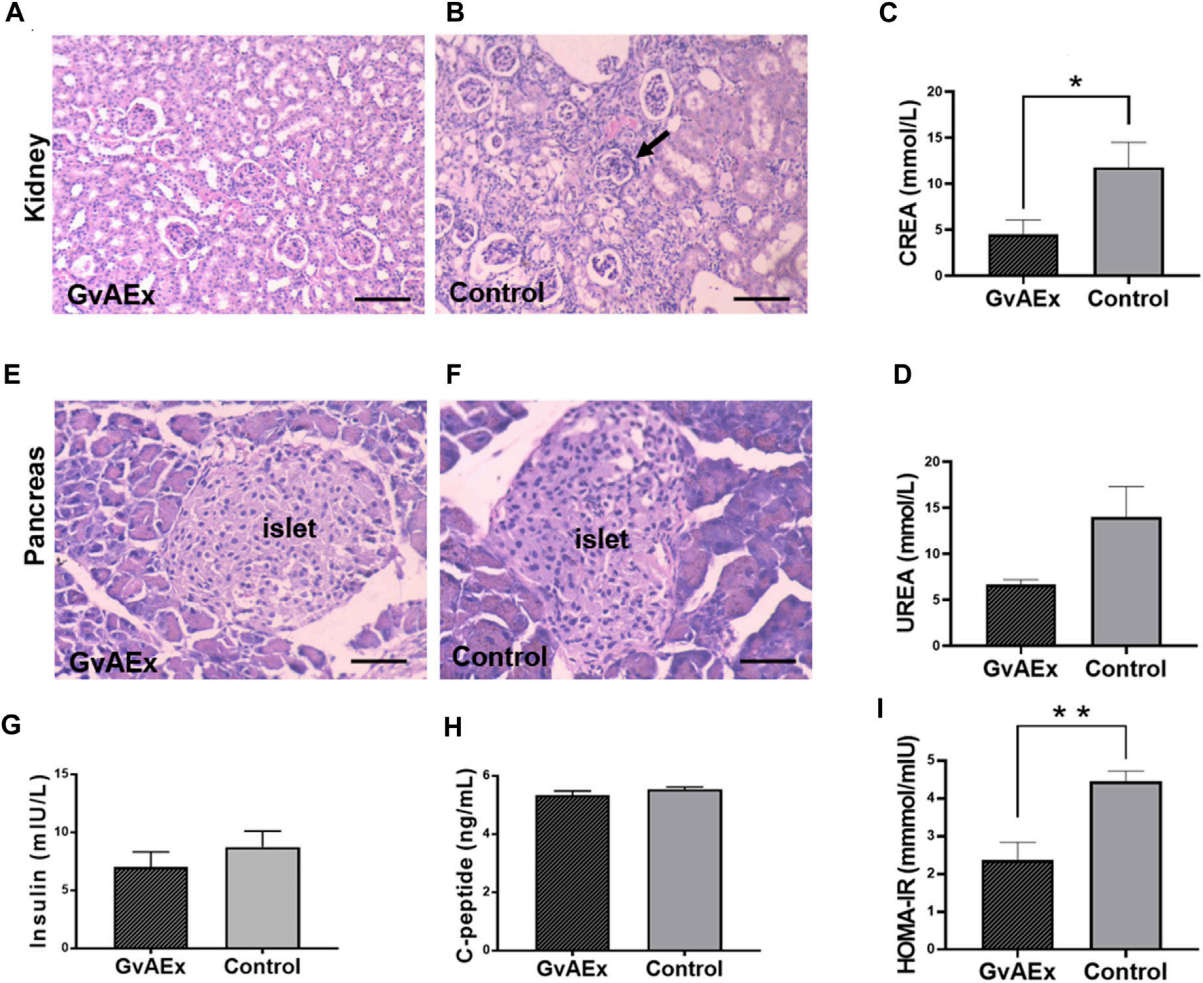
Treatment of GvAEx improves kidney and islet function in diabetic mice. **(A,B)** Representative light microscopic images of kidney after 12 weeks of GvAEx **(A)** or vehicle **(B)** treatment (scale bar = 100 μm). **(C,D)** Plasma levels of Creatinine (CREA) **(C)** and urea **(D)** in the db/db mice treated with GvAEx or vehicle for 12 weeks (*n* = 6/group). **(E,F)** Representative light microscopic images of pancreatic islet in GvAEx **(E)** and control mice **(F)** (scale bar = 50 μm). **(G,H)** Serum levels of insulin **(G)** and c-peptide **(H)** (*n* = 6/group). **(I)** HOMA-IR index of GvAEx and control mice (*n* = 6/group). *p < 0.05, **p < 0.01, ***p < 0.001.

To test the potential protective effects of GvAEx on diabetic nephropathy in db/db mice, we examined the histopathological features of kidney using hematoxylin-eosin (H-E) staining (Figures 2A,B). Diabetic kidney diseases are characterized by glomerular hyperfiltration, inflammation and fibrosis. Treatment with GvAEx showed less inflammatory cell infiltration and fibrosis in the kidney (Figure 2A) compared with control mice (Figure 2B). Consistent with less pathomorphologic features of diabetic nephropathy observed in the kidney, blood creatinine levels significantly decreased in the GvAEx-treated mice (Figure 2C; *p* < 0.05). There was also a trend of decrease in blood urea, but it did not reach statistical significance (Figure 2D).

In order to test whether glucose lowering effects of GvAEx were induced by improvement of islet function, we first detected morphological changes of the pancreatic islets. Islets sizes were comparable between the GxAEx-treated and control animals (Figures 2E,F). Additionally, fasting serum insulin concentrations were also comparable between the GvAEx-treated and control groups after 3 months of treatment (Figure 2G). C-peptide is a widely used measurement of pancreatic beta cell function. It reflects the amount of endogenous insulin secreted and maintained at a more constant rate over a longer period of time (Leighton et al., 2017). Likewise, we found no difference of fasting serum c-peptide concentrations between the GvAEx-treated and control groups (Figure 2H). HOMA-IR index is a widely used parameter that can estimate insulin resistance. GvAEx-treated mice displayed lower HOMA-IR index compared to those of the controls (Figure 2I *p* < 0.01). These results indicate that the glucose lowering effect of GvAEx administration might not be caused by increased insulin secretion from the pancreas but by improved insulin sensitivity in peripheral tissues.

### GvAEx Treatment Promotes Liver Function and Reduces Gluconeogenesis

**FIGURE 3:**
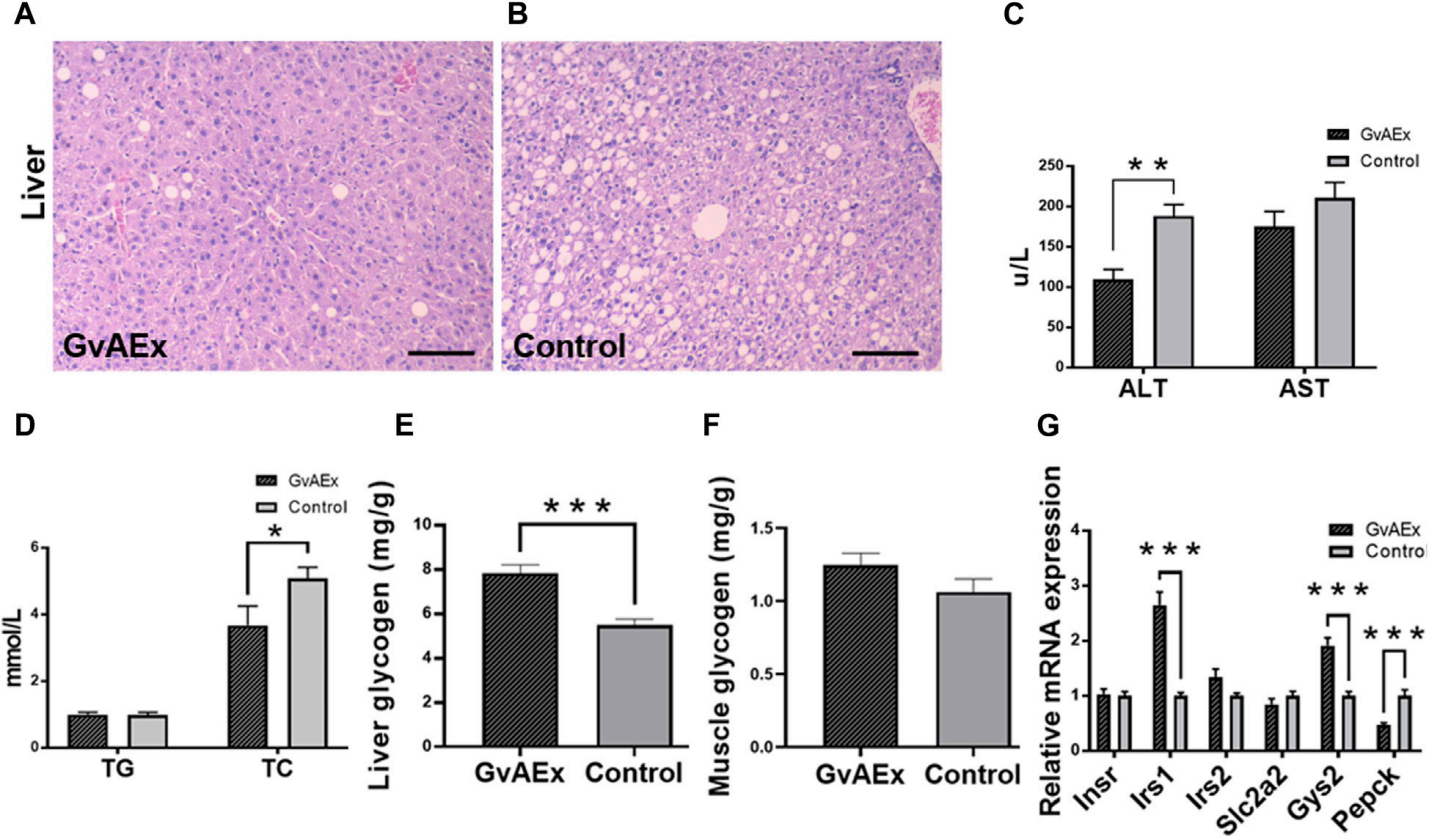
GvAEx treatment promotes liver function and reduces gluconeogenesis. **(A,B)** Representative light microscopic images of liver in db/db mice treated with GvAEx **(A)** or vehicle **(B)** for 12 weeks (scale bar = 100 μm). **(C)** Plasma levels of alanine aminotransferase (ALT) and aspartate transaminase (AST) (*n* = 6/group). **(D)** Plasma levels of total cholesterol (TC) and triglyceride (TG). **(E)** Hepatic glycogen content. **(F)** Skeletal muscle glycogen content (*n* = 6/group). **(G)** Relative mRNA expression levels of genes involved in glycogen synthesis and gluconeogenesis in the liver. *p < 0.05, **p < 0.01, ***p < 0.001.

The liver is one of the major peripheral targets for the action of insulin to regulate glucose homeostasis. The histopathological changes of liver obtained from gavage treatment of GvAEx or vehicle are shown in Figures 3A,B, respectively. Compared with the liver of vehicle control mice (Figure 3B) that showed foci of ballooning degeneration of hepatocytes, db/db mice treated with GvAEx displayed a relative normal liver histomorphology with fewer ballooning like structures (Figure 3A), indicating alleviated fatty liver conductions.

After 12 weeks of GvAEx treatment, there was a significantly decreased serum level of liver enzyme ALT (Figure 3C; *p* < 0.01) but not AST (Figure 3C). Plasma total cholesterol (TC) level significantly decreased in the GvAEx-treated mice compared to that of the vehicle controls (Figure 3D; *p* < 0.05), but triglyceride (TG) levels were comparable between GvAEx and control groups (Figure 3D).

Glycogen is primarily synthesized and stored in the liver and skeletal muscles (Prats et al., 2018). One of the key features of diabetes is dysregulation of liver glycogen metabolism, in which glycogen can be abnormally accumulated or depleted because of peripheral tissue insulin resistance. Therefore, we measured the proportion of glycogen in the liver to see if GvAEx had an impact on hepatic glycogen synthesis and storage. After 12 weeks of GvAEX gavage, glycogen levels in the liver dramatically increased compared to those of the control group (Figure 3E; *p <* 0.001). However, the skeletal muscle glycogen levels in db/db mice were comparable between the two groups (Figure 3F). Gene expression analyses have shown that in the case of Irs1 and Gys2, both genes are involved in insulin-regulated glycogen synthesis, were elevated in the liver after GvAEx treatment (Figure 3G; *p* < 0.001). In mRNA levels of phosphoenolpyruvate carboxykinase (Pepck), a gene plays a key role in the gluconeogenesis (Méndez-Lucas et al., 2013) and was significantly decreased in the GvAEx group (Figure 3G; *p* < 0.01), suggesting that GvAEx treatment could increase glycogen synthesis but reduce hepatic gluconeogenesis.

Taken together, our results have demonstrated that GvAEx treatment could ameliorate hepatic fat deposition and attenuate liver injury. In the meantime, GvAEx could increase gene expressions of hepatic glycogen synthesis while decreasing glucogenesis genes, as a result leading to the hypoglycemic effect in db/db mice.

### Effects of GvAEx on HepIR Cell Viability

To explore the effects of GvAEx *in vitro*, we treated mouse hepatic cell line HepIR with various doses of reagent. We first used CCK8 assay to measure cell viability and the cytotoxicity of GvAEx. The relative cell viabilities of HepIR treated with 0.1–3 mg/ml GvAEx for 24 h are shown in Supplementary Figure S2, and no significant inhibitions of cell viability were observed using GvAEx treatments with doses of up to 2 mg/ml when compared to vehicle controls. However, significant inhibitions of cell proliferation (29–41% inhibition) were observed starting at a dose of 2.2 mg/ml, and 3 mg/ml, the highest dosage used in the study (Supplementary Figure S2A). Therefore, we used 0.1–1.5 mg/ml GvAEx as safe dosage in the following *in vitro* studies.

### GvAEx Enhances Insulin Sensitivity and Glucose Uptake in Hepatocytes

As insulin resistance is a key pathophysiologic factor of T2DM, we mimic the insulin resistance-like condition *in vivo* by treating HepIR live cells with palmitic acid. The Supplementary Figure S2B showed that 200 μM palmitic acid significantly reduced phosphorylation of AKT, which implied successfully induced insulin resistance in HepIR cells. All subsequent cell experiments were based on this experimental condition.

**FIGURE 4:**
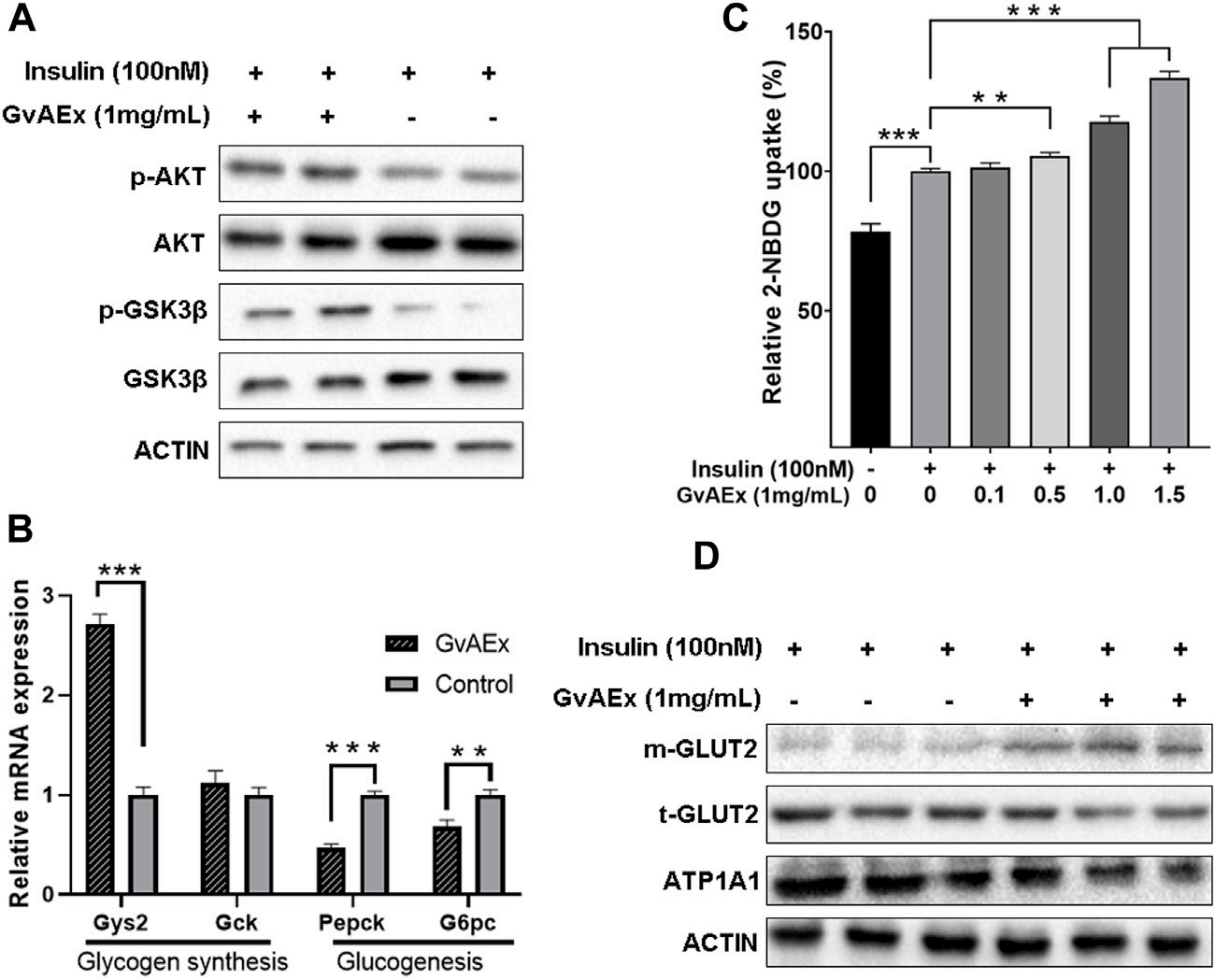
GvAEx enhances insulin sensitivity and glucose uptake in hepatocytes. **(A)** Western blot analysis of insulin signaling-related molecules in HepIR cells with or without GvAEx treatment. **(B)** Relative mRNA expression levels of genes involved in glycogen synthesis and gluconeogenesis in HepIR cells treated with or without GvAEx (*n* = 4). **(C)** Effects of GvAEx on glucose (2-NBDG) uptake of insulin-resistant HepIR cells (*n* = 6). **(D)**: Western blot analysis of total and membrane GLUT2. *p* *<0.05, **p < 0.01, ***p < 0.001.

To clarify whether and how GvAEx improved insulin sensitivity in hepatocytes, the expression and phosphorylation levels of insulin signaling-related molecules with and without GvAEx treatment were assessed. In contrast to the control group, GvAEx significantly increased the tyrosine phosphorylation level of AKT in HepIR cells (Figure 4A). GSK-3, a ubiquitously expressed serine/threonine protein kinase, is a critical downstream element of the insulin/PI3K/AKT pathway, whose activity can be inhibited by Akt-mediated phosphorylation at Ser9 of GSK-3β. GSK-3 can phosphorylate and inactivate glycogen synthase that leads to less glycogen synthesis (Srivastava and Pandey, 1998). We found that co-treatment with insulin and GvAEx (1 mg/ml) significantly increased the phosphorylation levels of GSK-3β when compared to insulin treatment alone (Figure 4A). What’s more, GvAEx treatment also up-regulated Gys2 mRNA expression levels but down-regulated Pepck level in HepIR cells (Figure 4B). Additionally, GvAEx decreased expression of G6pc, one of the glucose-6-phosphatase catalytic-subunit-encoding genes and a key enzyme in gluconeogenesis and glycogenolysis (Figure 4B). Our data has suggested that GvAEx treatment could activate insulin signaling, thus promoting glycogen synthesis while suppressing glucogenesis in the hepatocytes.

To determine changes in glucose uptake ability insulin-induced, we performed a fluorescent 2-NBDG (a fluorescence derivative of d-glucose) uptake assay. HepIR cells were treated with insulin (10 nM) alone or in the presence of GvAEx (0.1–1.5 mg/ml) and fluorescent 2-NBDG levels were monitored. The data has shown that insulin significantly increased 2-NBDG levels by up to 28.4% when compared to control cells (Figure 4C). In the presence of GvAEx there were significantly higher increased rates of 2-NBDG, whose effects were more pronounced with higher GvAEx concentrations, when compared to that of the insulin treatment alone (Figure 4C), suggesting that GvAEx could promote insulin-induced glucose uptake in a dose-dependent manner.

In the hepatocytes, GLUT2 is translocated from the cytoplasm to the plasma membrane in response to insulin and is the primary carrier for the transportation of extra-cellular glucose into the hepatocytes (Thorens, 2015). We have tested whether GvAEx had impacts on GLUT2 expression in hepatocytes. Serum-starved HepIR cells were treated with GvAEx (1 mg/ml) for 24 h. Serum-starvation and subsequent culture in the serum-free medium ruled out the possible influence of serum factors on the membrane translocation of GLUT2. We then extracted the whole cells and cell membrane proteins, respectively. The levels of total GLUT2 (t-GLUT2) did not change markedly between the groups (Figure 4D). However, compared to the insulin alone treatment group, insulin combined with GvAEx significantly increased the content of membrane GLUT2 (m-GLUT2), indicating that GvAEx could promote insulin induced GLUT2 translocation in hepatocytes.

Taken together, GvAEx treatment enhanced the expression of genes involved in hepatic glycogen synthesis and inhibited gene expression in gluconeogenesis. Additionally, it promoted glucose uptake and GLUT2 translocation from cytosol to membrane *in vitro*.

### GvAEx Treatment Alters the Composition of Microbiota in db/db Mice

**FIGURE 5:**
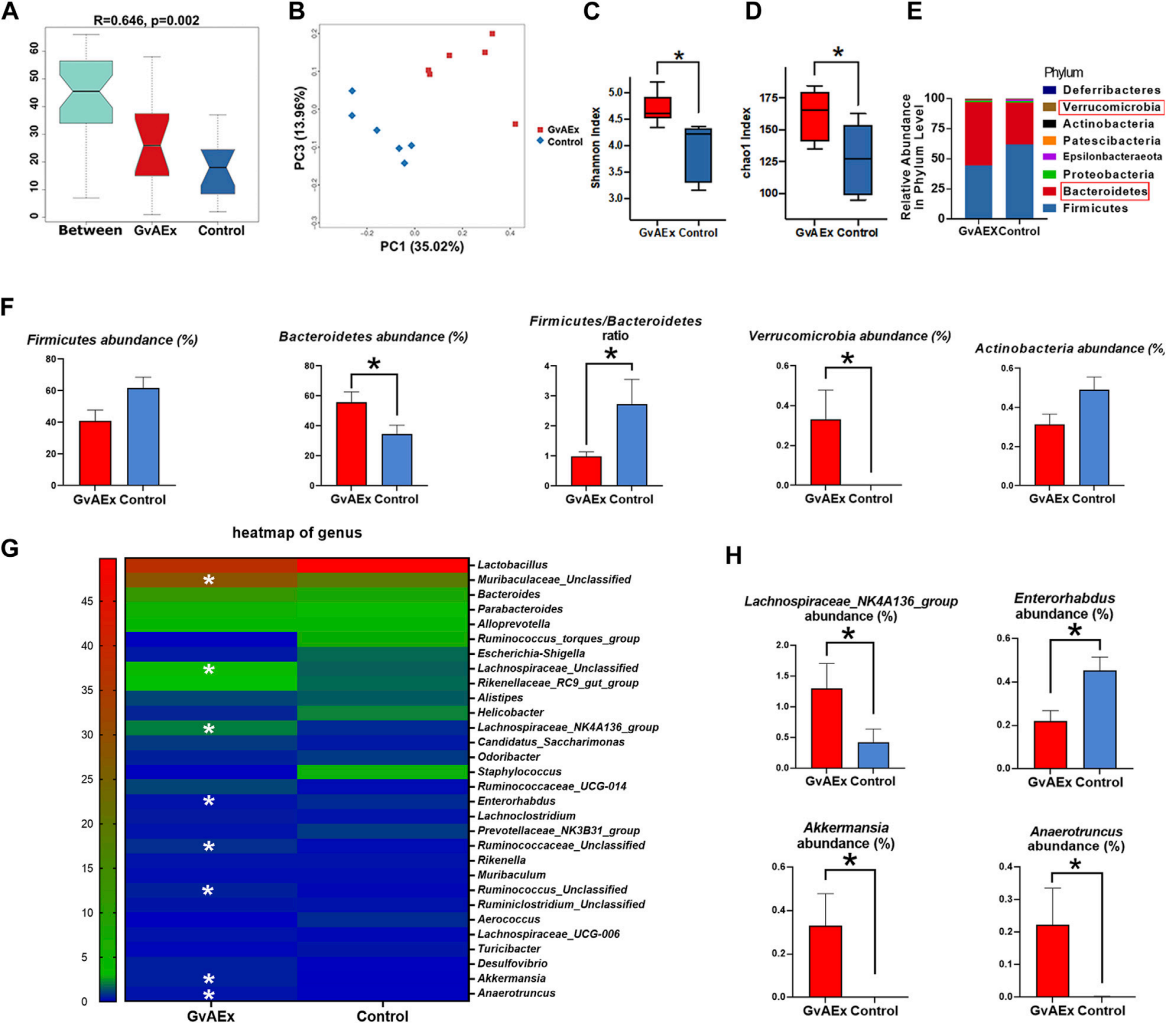
GvAEx treatment alters the composition of gut microbiota in db/db mice. (**A)** Anosim analysis of gut microbiota in db/db mice treated with GvAEx or vehicle for 12 weeks (*R* = 0.646, *p* = 0.002) (*n* = 6/group). **(B)** PCOA of gut microbiota in mice based on weighted UniFrac. **(C)** Species diversity indices (Shannon). **(D)** Species richness estimates (Chao1). **(E)** Phylum level comparison of gut microbiota proportional abundance in feces of GvAEx and control. **(F)** Relative abundance of microbiota phylum between the groups. **(G)** Heatmap of gut microbiota at the genus level in mice. **(H)** Relative abundance of microbiota at the genus level between groups. *p < 0.05.

In recent years, a large body of evidence has suggested that the gut microbiota may play an important role in the pathophysiology of obesity and diabetes (Qin et al., 2012; Wang et al., 2018; Gurung et al., 2020). Specifically, gut microbiota may mediate environmental factors related to glucose homeostasis by impacting major metabolic organs such as liver, muscle and fat (Gurung et al., 2020). Therefore, we have analyzed the composition of the intestinal flora of GvAEx oral gavage treated db/db mice using 16S rRNA sequencing. There were significant differences in colony distribution between the GvAEx treatment group and control group (Figure 5A, *R* = 0.646, *p* < 0.01). The PCoA analysis indicated that the gut microbiota structure in GvAEx-treated mice was distinctive from the untreated control group (Figure 5B). By estimating bacterial richness and calculated diversity, we have found that the GvAEx group had a significantly higher microbial diversity (Figure 5C, *p* < 0.05, Shannon) and number of microbial species (Figure 5D, *p* < 0.05, Chao1) than those of the control group.

The profound compositional changes of the gut microbiota were further analyzed in GvAEx-treated and vehicle group mice. At the phylum level, both *Firmicutes* and *Bacteroidetes* were the two most dominant phyla of the gut microbiota in GvAEx and control group (Figure 5E). However, GvAEx treatment significantly increased *Bacteroidetes* abundance when compared with that in the vehicle control (Figure 5F). Additionally, there were trends of decrease of *Firmicutes* abundance and *Actinobacteria* abundance in GvAEx group (Figure 5F). Meanwhile, the *Firmicutes* to *Bacteroidetes* ratio had also significantly decreased after GvAEx treatment (Figure 5F; *p* < 0.05). Furthermore, the phylum abundance of *Verrucomicrobia* increased when compared to that in vehicle control group (Figure 5F; *p* < 0.05).

At the genus level, we selected the top 30 abundant genera and generated a heatmap (Figure 5G). The data showed an increase in the abundance of Muribaculaceae*,* Lachnospiraceae*,* Lachnospiraceae*_ NK4A136_group,* Ruminococcaceae*, Ruminococcus, Akkermansia* and *Anaerotruncus* after GvAEx treatment while the abundance of *Enterorhabdus* was reduced significantly (Figures 5G,H). Taken together, these data suggest that GvAEx treatment significantly reshaped the gut microbiota composition of db/db mice.

## Discussion

*Psidium guajava* L. leaves have traditionally been widely used to treat several diseases, including infectious diseases, neoplasm, metabolic diseases and digestive diseases (Díaz-de-Cerio et al., 2017). There are several studies that have focused on elucidating the anti-diabetic compounds present in guava leaves. In particular, quercetin in the aqueous extract of guava leaves has been found to promote glucose uptake in hepatocytes and alleviate hyperglycemia in diabetes (Cheng et al., 2009). However, the underlying mechanism by which quercetin regulates the glucose uptake of hepatocytes is still unclear. Besides, studies have reported that guava leaf extract might exert its anti-diabetic effects by activating the PI3K/AKT signaling pathway in the liver and muscle of diabetic mice (Vinayagam et al., 2018; Jayachandran et al., 2020). Polysaccharides and flavonoid compounds purified from guava leaves synergistically inhibited *a*-glucosidase and *a*-amylase which would delay the absorption of glucose in the small intestine to lower blood glucose levels (Zhang et al., 2016; Beidokhti et al., 2020). Glucagon-like peptide-1 (GLP-1) is a hormone that lowers blood glucose levels by stimulating insulin, inhibiting glucagon, suppressing gastric emptying and promoting islet cell regeneration. Its’ degradation is controlled by dipeptidyl peptidase 4 (DPP4) (Smith et al., 2019). Ethanolic extract of guava leaves dose-dependently inhibited DPP4 due to individual flavonoid glycosides, such as quercetin, isoquercitrin, etc. (Eidenberger et al., 2013). The negative regulatory role of protein tyrosine phosphatase 1B (PTP-1B) in insulin signaling prevents insulin receptors from binding to insulin, which in turn causes insulin resistance and ultimately leads to T2DM. Methanol extract of *Psidium guajava* leaves was found to possess significant inhibitory effect on PTP1B (Oh et al., 2005). However, all of the above studies only confirmed the inhibitory effects of phytocompound of guava leaf on the related enzyme activities *in vitro*. We have previously reported that GvAEx is rich in flavonoids, which may partially contribute to its anti-diabetic effects (Bai et al., 2019); however, the underlying molecular mechanism is unclear. In the present study, we’ve provided evidence demonstrating that the total extract of guava leaves, GvAEx, could be used as an anti-diabetic agent by modulating hepatic glucose metabolism and altering the intestinal flora composition.

In our study, we have discovered that GvAEx significantly reduced FPG levels in T2DM mice in a dose-dependent manner. In a previous study, oral administration of guava leaf extract had beneficial anti-obesity effects on SHRSP. Z-Leprfa/IzmDmcr rats (Yoshitomi et al., 2012). However, inconsistent with this previous study, we did not observe reduced obesity after GvAEx administration in the db/db mice. This could be due to the different genetic background of the animal models used in the studies. Additionally, GvAEx could improve glucose tolerance and insulin sensitivity in db/db mice. These results suggested that GvAEx gavage have beneficial effects on glucose metabolism in diabetic mice.

It is well established that certain natural extracts such as ginseng could increase insulin secretion to lower plasma glucose in animals and humans (Lee et al., 2006). In our study we found no difference in serum insulin levels and c-peptide levels between the two groups, thus we proposed that the glucose lowering effects of GvAEx were not due to promotion of insulin secretion, but to improve insulin sensitivity in the peripheral tissues.

The liver is the major site for glucose and lipid metabolism, and hepatic insulin resistance is thought to be one of the main causes of fasting hyperglycemia (Petersen and Shulman, 2018). In the current study, we’ve discovered that not only did GvAEx treatment alleviate fatty liver morphological conditions, it also improved liver insulin sensitivity *in vivo* and *in vitro*. Compared to the control group, GvAEx treatment significantly lowered HOMA-IR values, which is an indicator of improved liver insulin sensitivity. It is well established that insulin sensitivity can be regulated directly or indirectly by modulating the components of the insulin signaling pathway, such as insulin receptor (IR), insulin receptor substrates (IRS) and AKT (Yang et al., 2018). The insulin-bound IR promotes the binding and activation (Tyr phosphorylation) of IRS after insulin activation, which then activates the phosphoinositide 3-kinase (PI3K)/AKT pathway (Huang et al., 2018; Yang et al., 2018). AKT plays a pivotal role in the regulation of various biological processes, including apoptosis, proliferation and intermediary metabolism. We’ve discovered that GvAEx significantly increased AKT phosphorylation levels in HepIR cells compared with the control group, suggesting that improved insulin sensitivity might be caused by increased insulin signaling activity in hepatocytes.

Besides improvement of insulin sensitivity, the GvAEx treatment also regulated glycogen synthesis and gluconeogenesis in hepatocytes. Under physiological conditions, insulin lowers blood glucose levels by prompting the liver and muscles to take up glucose from the blood and store it as glycogen. GSK-3 could be inhibited upon phosphorylation *via* AKT, which results in an increase of glycogen synthesis (Sesti, 2006). Additionally, the liver contributes significantly to maintaining the balance of glucose metabolism by altering the levels of hepatic glucose release, controlling the processes of gluconeogenesis and glycogenolysis (Hatting et al., 2018). Our results have shown that the GvAEx treatment elevated levels of phosphorylation of GSK-3β in HepIR cells, which were consistent with increased hepatic glycogenesis *in vivo* and *in vitro*. In the meantime, the mRNA levels of Pepck and G6pc was significantly lower in the GvAEx group, suggesting a suppressive effect on gluconeogenesis in the hepatocytes.

Glut-2 is the principal transporter for transfer of glucose between liver and blood. It mediates the amount of glucose in and out of hepatocytes by altering the rate of glucose uptake in hepatocytes (Thorens, 2015). Insulin stimulation could promote the translocation of GLUT2 from the cytosol to the plasma membrane, which leads to increased glucose uptake in hepatocytes (Ding et al., 2016). In the present study, we’ve discovered that GvAEx administration could facilitate glucose uptake by promoting insulin-induced translocation of GLUT2 from the cytosol to the membrane, while not affecting overall GLUT2 level in HepIR cells, which might partly contribute to in the hypoglycemic effects of GvAEx treatment in db/db mice.

In previous studies, plant flavonoids and terpenoids were widely used in prevention of obesity and diabetes by ameliorating insulin resistance, specifically, by activating AMPK and PI3K/AKT signaling (Ding et al., 2012; Zhang et al., 2012; Paoli et al., 2013; Rodríguez-Rodríguez et al., 2015). Therefore, the pluripotent effects of GvAEx on hepatic glucose metabolism and glycogen might be due to its richness in terpenoids and flavonoids. Based on these previous studies, we’ve suggested that the multiple bioactive components present in GvAEx synergistically exerted anti-diabetic effects by improving insulin resistance, promoting glycogen synthesis, inhibiting hepatic gluconeogenesis and enhancing glucose uptake in the hepatocytes.

Numerous studies have noted the important role of gut microbiota in the development and treatment of T2DM (Xu et al., 2015; Bauer et al., 2018; Zhao et al., 2018). In particular, the gut microbiome can interact with dietary components and habits to influence host insulin sensitivity, intestinal permeability, and glucose and lipid metabolism (Gurung et al., 2020). Both first-line drug metformin and traditional Chinese medicine can significantly alter the composition of the gut microbiota, thus helping to ameliorate hyperglycemia (Xu et al., 2015; de la Cuesta-Zuluaga et al., 2017; Wang et al., 2017; Bauer et al., 2018). Several studies have shown that the gut microbiota of obese and diabetic animals and humans exhibit a higher *Firmicutes*/*Bacteroidetes* ratio compared with normal individuals, and suggested that this ratio could be used as a reliable biomarker of impaired glucose metabolism (Ley et al., 2006; Yue et al., 2019). However, no studies have explored the possible mechanisms of the hypoglycemic effects of GvAEx from the perspective of the gut microbiota. In the present study, for the first time, we reported that there were significant differences in microbiota composition between the GvAEx and control groups. GvAEx treatment significantly increased the abundance and diversity of intestinal flora in db/db mice. Besides, the GvAEx group exhibited less *Firmicutes* abundance but richer *Bacteroidetes* abundance. The *Firmicutes*/*Bacteroidetes* ratio also decreased significantly after GvAEx treatment, indicating an improvement of glucose homeostasis. Bacteroidetes mainly produce propionate, which may reach the colon and stimulate the secretion of GLP-1, thus producing a hypoglycemic effect (Chambers et al., 2015). However, we did not detect plasma GLP-1 level in our study, which would be an interesting investigation in the future. *Bacteroides* and *Alstipes* are positive outcome predictors for metabolic diseases, such as obesity and diabetes (Kuang et al., 2017; Zhang et al., 2021). In the present study, we’ve found that the relative abundance of *Bacteroides* and *Alstipes* was higher in GvAEx group, albeit not statistic significantly, probably due to the limited sample size. We’ve also found that GvAEx treatment significantly reduced relative abundance of *Enterorhabdus* compared to the control group. Inflammation plays a very important role in the development of insulin resistance. Currently, T2DM is considered to be a chronic inflammatory disease. In many studies related to intestinal inflammation, a significant increase in the abundance of *Enterorhabdus* has been observed (Tang et al., 2021; Wang et al., 2021). It was also found that the abundance of *Enterorhabdus* was significantly increased in non-alcoholic fatty liver disease (NAFLD) mice (Li et al., 2021). In addition, *Enterorhabdus* have been found to be associated with T2DM and enterotoxicity (Shibayama et al., 2019). Short chain fatty acid (SCFA) was reported to be beneficial in regulating the functions of adipose tissues, skeletal muscles and the liver, thus contributing to improved glucose homeostasis and insulin sensitivity (Canfora et al., 2015; Zhang et al., 2015; Ma et al., 2020; Xu et al., 2020). The decrease in circulating SCFA may have an important role in the development of insulin resistant and diabetes (Saad et al., 2016). Importantly, SCFA plays a key role in glucose homeostasis due to its capacity to increase insulin sensitivity and resist inflammation (Säemann et al., 2000; Gao et al., 2009). Although we did not measure the intestinal SCFA level in the present study, the abundance of the Lachnospiraceae *NK4A136 group* (a SCFA-producing bacterium) and other beneficial bacteria *Akkermansia* and *Anaerotruncus* were significantly higher in the GvAEx-treated group. *Akkermansia* are thought to be associated with the amelioration of metabolic diseases and inflammation due to their anti-inflammatory and insulin-sensitive properties (Graessler et al., 2013). *Akkermansia* are able to degrade mucins and protect the intestinal mucosal layer (Zhang et al., 2019). These changes in these SCFA-producing-related bacteria may partially explain the ameliorated insulin resistance and improved lipid profiles in db/db mice treated with GvAEx.

**FIGURE 6:**
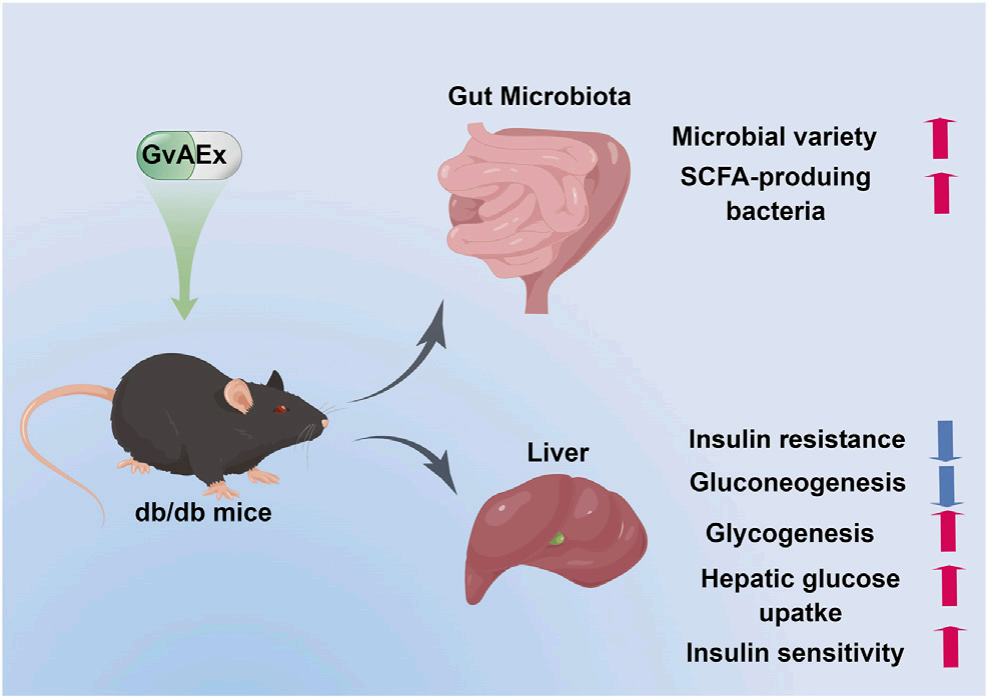
Proposed working model of GvAEx induced anti-diabetic effect in db/db mice.

To summarize, in this study, we have demonstrated the anti-diabetic functions and underlying mechanisms of GvAEx in db/db mice. As shown in Figure 6, GvAEx might improve insulin sensitivity in the liver by promoting hepatic glycogen synthesis and inhibiting hepatic gluconeogenesis *via* modulating insulin-related signaling pathways. We also found that GvAEx could elevate the expression of GLUT2 on the cell membrane of hepatocytes, which might promote the uptake of glucose by hepatocytes, leading to an improved glucose metabolism. More importantly, for the first time, we reported that GvAEx might also alter the composition of the gut microbiota and increase the enrichment of probiotics, thus exerting its sustained beneficial effects on glucose metabolism. GvAEx has the potential for drug development against T2DM.

### Limitations and Future Investigations

In this study, GvAEx from Guava leaf aqueous extracts were used for their hypoglycemic effect as a compound formulation. The exact ingredients or class of ingredients that exerted their effects on the db/db mice were not clear and deserve further exploration in future studies. However, we revealed profound compositional differences in gut microbiota between GvAEx treatment and control group. Further metagenomic sequencing needs to be done to explore the functional diversity of microbial communities between the two groups. Besides, we only tested several indicators of hepatic and renal toxicity. The comprehensive toxicological studies of the exacts should be conducted in the future.

## Data Availability

The datasets presented in this study can be found in online repositories. The names of the repository/repositories and accession number(s) can be found below: https://www.ncbi.nlm.nih.gov/bioproject/, PRJNA820451.
