# Supplementary figures and images for: Aqueous Extract of Guava (Psidium guajava L.) Leaf Ameliorates Hyperglycemia by Promoting Hepatic Glycogen Synthesis and Modulating Gut Microbiota
Source: Front Pharmacol. 2022 Jun 1;13:907702. doi: 10.3389/fphar.2022.907702 (PMC9198539; doi:10.3389/fphar.2022.907702)

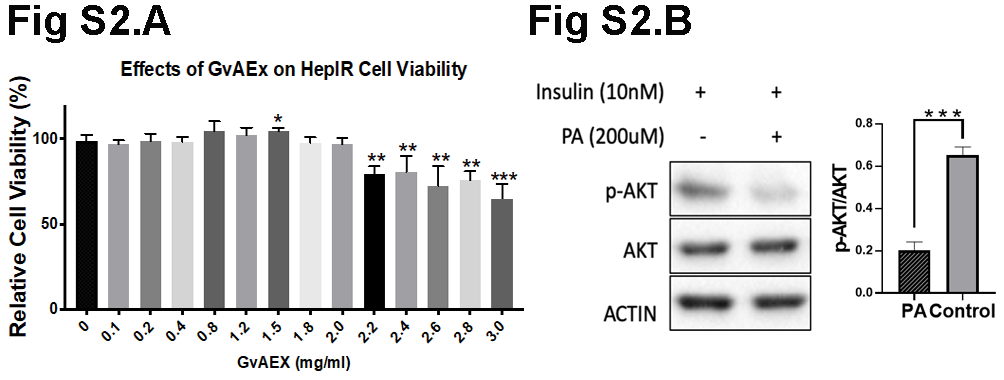

Supplement: Supplementary file 2 [file Image2.TIF]

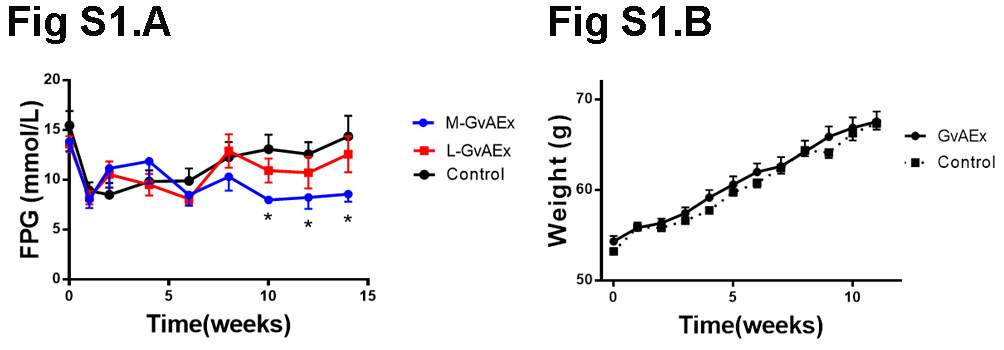

Supplement: Supplementary file 3 [file Image1.TIF]
